# Financial transfers from adult children and depressive symptoms among mid-aged and elderly residents in China - evidence from the China health and retirement longitudinal study

**DOI:** 10.1186/s12889-018-5794-x

**Published:** 2018-07-16

**Authors:** Yue Wu, Wanyue Dong, Yongjian Xu, Xiaojing Fan, Min Su, Jianmin Gao, Zhongliang Zhou, Louis Niessen, Yiyang Wang, Xiao Wang

**Affiliations:** 1 School of Public Policy and Administration, Xi’an Jiaotong University, Xi’an, People’s Republic of China; 2 School of Public Health, Center of Medical Science, Xi’an Jiaotong University, Xi’an, People’s Republic of China; 3 Liverpool School of Tropical Medicine, Pembroke Place, Liverpool, UK; 4 International Business School Suzhou, Xi’an Jiaotong-Liverpool University, Suzhou, Jiangsu People’s Republic of China

**Keywords:** Depressive symptoms, Mid-aged and elderly residents, Multilevel model, CHARLS, China, Inter-generational transfer

## Abstract

**Background:**

Although the awareness of mental health problems in late life is rising, the association between financial transfers to the older generations from children and mental health at older ages in China has received little attention. This study examines the association between financial transfers from children and depressive symptoms among the mid-aged and elderly residents (from 45 years of age and older) in China.

**Methods:**

We used the data from the China Health and Retirement Longitudinal Study (CHARLS, 2013) (*n* = 10,935) This included data on financial transfers from all non-co-resident children to their parents, and the individual scores on depressive symptoms as measured by the 10-item Center for Epidemiologic Studies–Depression Scale (CESD-10). A two-level - individual and community levels - mixed linear model was deployed to explore their association.

**Results:**

Financial transfers from children to parents was the major component of inter-generational financial transfers in Chinese families. A higher financial support from non-co-resident children was signivicantly and positively related to fewer depressive symptoms (coef. = − 0.195,*P*-value< 0.001) among both the mid-aged and elderly parents.

**Conclusions:**

Financial transfers from non-co-resident children are associated with depressive symptoms among mid-aged and elderly residents in the China situation. Taxation and other policy measures should encourage and facilitate these type of financial transfers and prevent a decrease of support from children to parents.

**Electronic supplementary material:**

The online version of this article (10.1186/s12889-018-5794-x) contains supplementary material, which is available to authorized users.

## Background

Depression, one of the most common mental disorders, affects approximately 7% of the world’s elders [1]. Almost 30% of males and 43% of females among the mid-aged and elderly residents in China reportedly suffered from depressive symptoms [2], as measured by 10-item Center for Epidemiologic Studies–Depression Scale (CESD-10), a widely used tool for studying mental health or depression

Inter-generational transfer also is named as inter-generational exchange [3] or inter-generational support [4], which may flow upwards from offspring to their (grand) parents or flow downwards from the elderly to their offspring. The transfers include financial transfers, instrumental transfers, emotional transfers and other kinds of support between family members [5–7].Inter-generational transfers have previously been found contributing to psychological well-being of elders [8].

It is well-known that family support plays an extremely important role in residents’ late life in Chinese traditional culture [9]. However, little research has explored the relationship between inter-generational transfers and mental health [3, 10]. A finding in Anhui Province of China showed that, emigration enhanced the amount of financial transfers from non-co-resident children, and the transfer had a positive effect on psychological outcomes among parents living alone [11]. Meanwhile, a finding among female elders in rural China showed that receipt of financial transfers from their children was negatively associated with subjective health [4]. So far there is no research based on nationally representative data, the research results were inconsistent and the extent of transfers and depressive symptoms were seldom measured [12, 13]. Moreover, family networks and community relationships which likely are significantly associated with mental health [14–16], have hitherto seldom studied.

Therefore, using a nationally representative data, our study aims to examine whether financial transfers – both money support and in-kind support - from non-co-resident children are associated with the parents’ depressive symptoms in the China situation. The extent to which the financial transfers were received and number of depressive symptoms were measured, and the community-level effect was involved in the analysis as well. This study may broaden our understanding of the relationship between financial transfer and mental health among elderly Chinese parents, and inform policy making.

## Methods

### Data and sampling

Data were from the second wave of China Health and Retirement Longitudinal Study(CHARLS). The baseline of CHARLS was designed based on the Health and Retirement Study (HRS), the English Longitudinal Study of Ageing (ELSA), the Survey of Health, Ageing and Retirement in Europe (SHARE), and similar longitudinal aging surveys in other countries. The second wave of CHARLS used in this study was held in 2013, which covered 445 villages\communities in 150 counties\districts within 28 provinces, where a multistage stratified sampling strategy and Probability-Proportional-to-Size Sampling(PPS) were adopted to sample participants aged 45 years old and above [17]. In the case of household sampling, all the buildings in each Primary sampling unit (PSU) were photographed and GPS positioned, and the households in each building were listed. Among them, collective residences such as military, schools, dormitories and nursing homes are excluded from the sample set. Accordingly, participants (*n* = 18,605) in CHARLS (2013) are all non-institutional.

CHARLS includes questions involving various aspects of mid-aged and elderly residents’ lives, including transfers among family members and depressive symptoms measured by 10-item Center for Epidemiologic Studies–Depression Scale (CESD-10) [18]. Our sample framework include non-institutional respondents with complete data on the financial transfers (> 0 RMB) from their own children, depressive symptoms and other major possibly related co-variables.

### Measurement of depressive symptoms

A Chinese version 10-item short form of the Center for Epidemiologic Studies–Depression Scale (CESD-10) was used to assess respondents’ depressive symptoms in CHARLS, and an additional file shows this in more detail [see Additional file 1]. The scale has been shown to have good reliability and validity (Cronbach’s α = .81) [19].

In our study, scores of 10 items were calculated as the value of dependent variable [2, 20]. The 10 items consisted of eight negatively oriented and two positively oriented questions. The negatively oriented ones included statements like “I was bothered by things that don’t usually bother me” and the positive ones are “I felt hopeful about the future” and “I was happy”. Each item was assessed by a four-point scale, scored as follows: 0 = Rarely or none of the time (< 1 day), 1 = Some or a little of the time (1–2 days), 2 = Occasionally or a moderate amount of the time (3–4 days), and 3 = Most or all of the time (5–7 days). The two positively oriented items were scored inversely. The CESD-10 score ranged from 0 to 30; the higher the scores, the more depressive symptoms a participant has.

### Measurement of inter-generational financial transfers

Financial and in-kind support from non-co-resident children in the past 1 year were used to assess financial transfers. The sum value of all children’s supports was calculated, and the logarithm of the sum was taken as independent variable value as in a related study [21]. The economic supports including total money support and total in-kind support, calculated in Chinese Renminbi Yuan (1 yuan = 0.1582 US dollars). Total money supports include living expenses such as water, electricity, telephone rates and loans, as well as other - regular and irregular - costs. Total in-kind supports include food, clothes, and other regular and irregular material support.

### Measurement of control variables

Other factors that may affect inter-generational transfers and depressive symptoms were included in the analysis as well. The control variables were grouped into four categories: other transfers, demographic characteristics, socio-economic characteristics, and current health status and social activity variables.

The specific variables of other transfers included: support for care of grandchildren, indicating whether grandchild care was provided, coding as 0 (not taking care of grandchildren) and 1 (taking care of grandchildren) [22]. Frequency of contact with children was measured by how often did they contact with each child, coding as 0(seldom, more than once a year), 1(sometimes, more than once a week and less than once a year), and 2(often, less than once a week). The means of the contact included phone call, text message, mail, or email. Living arrangement indicated whether one respondent lived with any child, coding as 0 (no) and 1(yes).

Demographic characteristics included: gender (0 = male;1 = female), marital status (0 = other;1 = living with a spouse), and age (were grouped into 4 groups based on the quartiles: 0 = 45–56 years old;1 = 57–62 years old;2 = 63–69 years old;3 = 70 years and older). Socioeconomic characteristics included: Education (continuous variable), ranging from 0 (illiterate) to 10 (Master’s degree). Income, measured by net household expenditure per capita, where net household expenditure was calculated as total household consumption expenditure [23], and the logarithm of net household expenditure per capita was deployed to assess the income level. Community development level, assessed by the summed number of amenities/associations. Geographical divisions of China, ranging from 0 to 6 (0 = Northwest (worst economic development); 1 = Southwest; 2 = Northeast; 3 = East; 4 = South; 5 = Central; 6 = North). Urban and rural areas were coded as 0 (rural) and 1(urban).

Health and social factors included: Activities of daily living (ADL), for which we asked the respondents whether have any difficulties with activities of daily living (dressing/bathing/eating/getting (in/out of) bed/using the toilet) (0 = no; 1 = yes). The sum of diagnosed chronic disease ranged from 0 to 14 (e.g. hypertension, diabetes or high blood sugar). The respondents were also asked whether they participated in any social activities (e.g. interacted with friends, played Ma-jong, or went to community club) (0 = no; 1 = yes), and whether they were at work (0 = no; 1 = yes).

### Statistical analysis

As the CHARLS data were collected based on multistage stratified sampling, and community was significantly associated with mental health, a 2-level mixed linear model [24] was conducted to assess the relationship between receipt of financial transfers and depressive symptoms. The first level is individual level (*n* = 10,935), and the second level is community level (*n* = 445). STATA12.0 was used for the statistical analysis.

## Results

### Characteristics of study population

The proportion of elder adults receving financial support from their children is 61.5. Table 1 presents the included characteristics of the respondents who receving financial support from their children in the study. The sample size of this study was 10,935. The mean value of CESD-10 score and logarithm of inter-generational financial transfers were 7.3 (SD = 6) and 7.5 (SD = 1.3), respectively.

**Table 1:** Descriptive statistics of the study sample (*N* = 10,935)

Variables		Mean (%)	SD
Dependent variable	Depressive symptoms (cesd-10^a^ score) (0–30)	7.3	6
Independent variable	Inter-generational financial transfers (log of transfers)	7.5	1.3
Other transfers	Whether take care of grandchildren		
	No (ref.)	58.1%	
	Yes	41.9%	
	Contact with children		
	Seldom(ref.)	37.4%	
	Sometimes	34.5%	
	Often	28.1%	
	Living with any child		
	No(ref.)	56.7%	
	Yes	43.3%	
Demographic factors:	Age group		
	45–56(ref.)	23.7%	
	56–62	24.9%	
	62–69	25.4%	
	69-	26.0%	
	Gender		
	Male (ref.)	46.8%	
	Female	53.2%	
	Marital status		
	Other (ref.)	14.0%	
	Living with spouse	86.0%	
Socioeconomic factors:	Education level (1–10)^b^	3.1	1.9
	Income level	8.6	1
	Amenities/associations (0–14)^c^	3.1	3.4
	Areas of china		
	Northwest (ref.)	6.1%	
	Southwest	18.9%	
	Northeast	7.1%	
	East	23.9%	
	South	8.4%	
	Central	22.4%	
	North	13.2%	
	Rural-urban		
	Rural (ref.)	66.3%	
	Urban	33.7%	
Health and social factors:	Any difficulty with ADL^d^		
	No (ref.)	92.8%	
	Yes	7.2%	
	Chronic disease (0–10)^e^	0.4	0.8
	Whether participated in any social activities		
	No (ref.)	39.6%	
	Yes	60.4%	
	Whether still on work		
	No (ref.)	35.0%	
	Yes	65.0%	

*SD* = standard deviation

^a^ CEDS-10: Center for Epidemiologic Studies Depression Scale (Range 0–30, Mean = 7.3, SD = 6.0)

^b^ Education level: including 10 levels,1 = No formal education (illiterate);2 = Did not finish primary school but capable of reading and/or writing;3 = home school;4 = Elementary school;5 = Middle school;6 = High school;7 = Vocational school;8 = Two-/Three-Year College/Associate degree;9 = Four-Year College/Bachelor degree;10 = Master degree (Range1–10, mean = 3.1, SD = 1.9)

^c^ Amenities/associations (0–14): Summed number of amenities/associations, was used to assess community building level, which was significantly associated with the mental health. (Rang 0–14, Mean = 3.1,SD = 3.4)

^d^ ADL: activities of daily living (dressing, walking, bathing, eating, getting in/out of bed, toileting, and experiencing incontinence)

^e^ Chronic disease: summed number of diagnosed chronic disease (hypertension, Dyslipidemia, diabetes or high blood sugar, Cancer or malignant tumor, Chronic lung diseases, Liver disease, Heart attack, Stroke, Kidney disease, Stomach or other digestive disease, Stomach or other digestive disease, Stomach or other digestive disease, Arthritis or rheumatism, Asthma) (range0–14, Mean = 0.4, SD = 0.8)

In terms of control variables, the average age was (62.7 ± 9.13) years old. Females accounted for almost half of the sample (46.8%). Among the respondents, less than half of them lived with their child (43.3%), and more than four fifths of them lived with spouse (86.0%). Respondents from rural areas (66.3%) were almost twice of whom from urban areas (33.7%). Very few of the respondents had difficulty with ADL (7.2%), and almost two-thirds of the respondents remained at work (65%).

### Inter-generational transfers and depressive symptoms

Results of the mixed-effects multilevel regression analysis are presented in Table 2. The results indicate that there was a significantly negative correlation between inter-generational financial transfers from children and depressive symptoms among the mid-aged and elderly residents in China (*P* < 0.001). The relationship was controlled by the effects of other variables such as other transfers with children, demographic factors, socioeconomic factors, and health and social factors.

**Table 2:** Mixed-effects ML regression results

Variables		B	SE	95% CI	
				Low	High
Independent variable	Inter-generational financial transfers (log of transfers)	−0.226^c^	0.044	−0.314	− 0.140
Other transfers	Whether take care of grandchildren				
	No (ref.)				
	Yes	0.286^a^	0.117	0.057	.516
	Frequency of contact with children				
	Seldom (ref.)				
	Sometimes	−0.071	0.132	−0.329	0.187
	Often	− 0.293^a^	0.142	− 0.572	− 0.014
	Living with any child				
	No (ref.)				
	Yes	−0.363^b^	0.116	− 0.590	− 0.134
Demographic factors:	Age group (years old)				
	45–56 (ref.)				
	56–62	0.269	0.162	−0.048	0.586
	62–69	0.448^b^	0.167	0.122	0.775
	69-	−.761^c^	0.189	−1.131	−0.391
	Gender				
	Male (ref.)				
	Female	1.547^c^	0.120	1.311	1.781
	Marital status				
	Other (ref.)				
	Living with spouse	−0.649^c^	0.170	−0.983	−0.315
Socioeconomic factors:	Education level (1–10)	−0.087^a^	0.035	− 0.1561	− 0.018
	Income level	− 0.170^b^	0.059	− 0.285	−0.056
	Amenities/associations (0–14)	−0.072^b^	0.028	−0.126	− 0.018
	Areas of china				
	Northwest (ref.)				
	Southwest	−0.411	0.357	−1.110	0.288
	Northeast	−2.414^c^	0.422	−3.241	− 1.586
	East	−1.994^c^	0.348	−2.675	− 1.313
	South	−1.267^b^	0.403	−2.058	−0.478
	Central	−1.307^c^	0.350	−1.994	−0.621
	North	−1.420^c^	0.380	−2.165	−0.675
	Rural-urban				
	Rural (ref.)				
	Urban	−0.546^b^	0.202	−0.941	− 0.151
Health and social factors:	Any difficulty with ADL				
	No (ref.)				
	Yes	1.595^c^	0.220	1.164	2.026
	Chronic disease (0–10)	0.259^c^	0.065	0.131	0.387
	Whether participated in any social activities				
	No (ref.)				
	Yes	−1.695^c^	0.115	−1.920	−1.470
	Whether still on work				
	No (ref.)				
	Yes	−0.065	0.136	−0.332	0.203
	_cons	13.203^c^	0.896	11.855	14.551

*ML* = Multi-level; *B* = unstandardized coefficient; *SE* = Standard error; *CI*: Confidence interval

^a^*p* < 0.05, ^b^*p* < 0.01, ^c^*p* < 0.001

Table 2 also indicates the associations between control variables and depressive symptom. We found the risk factors of depressive symptoms including taking care of grandchildren, aged 62–69 years old, being females, having difficulty with ADL, being diagnosed with more chronic diseases, as well as protective factors including living with a child or spouse, aged 69 years old and over, living in better developed or urban areas, higher levels of education, income and community development, participating in social activities.

## Discussion

This is the first study reporting the inverse association between extent of financial transfers and depressive symptoms among the mid-aged and elderly Chinese and is based on a large-scale, nationally representative survey (CHARLS) in China. It provides convincing evidence that a higher amount of financial support from non-co-resident children is positively related to less depressive symptoms among their older Chinese parents.

### Inter-generational financial transfers in China

In Europe, inter-generational financial transfers are in the opposite direction i.e. mainly from parents to children [5, 8, 25]. Our study shows that economic resources between parents and adult children flow mainly from children to parents (*n* = 10,935), while it finds a very small number of transfers from parents to children. Additionally, compared to the amount of elders’ received financial support, the amount given to their adult children was relatively small in China [4, 10, 21, 26]. That is in line with the transfer direction in some Asian countries, such as in Korea and Philippines [26, 27]. We did not include financial transfer from parents to children in our analyses, as the number of transations and the amount per transaction are very low.

Factors connected with the specific configuration of welfare regimes are relevant to inter-generational transfers [5]. The particular pattern and direction of inter-generational transfer in China could be interpreted by the following factors connected with the specific configuration of welfare regimes, like those in relation to ‘family policy’ [28, 29]. In China, children hold obligations to support their parents [30], and the adult children should support their elderly parents financially and ensure their basic living needs [31]. The accelerated emigration as well as inadequate pension and health insurances for the elderly, especially in China’s rural areas, made it particularly urgent to oblige non-co-resident children to support their parents financially [32]. Additionally, Chinese traditional culture of “filial piety is the foundation of all virtues” may play an important role in cross-generational transfer between children and parents [33].

### Inter-generational financial transfers and depressive symptoms

Higher levels of economic resources and better health are show to be positively associated in other studies. [34, 35]. In relation to inter-generational financial transfers, Jewish Israelis and Coreans showed that the effect of financial support from children was not significant assocated to reduce depression [3, 27]. This is in contrast to our study findings that show a increased financial support from non-co-resident children positively related to less depressive symptoms among older parents. This is similar to findings in Hong Kong and Taiwan [10].

Findings for other studies may explain the positive association that higher amount of receipt of financial transfer from non-co-resident children and fewer depressive symptoms among mid-aged and elderly parents in China. When there is internal emigration by working-age adults from rural to urban and from western to eastern China, fewer old parents live with children, we found that less than half (41.9%) of the older parents lived with one of their children. Thus, for adult children, financial transfers may be a kind way to compensate the effects of living apart from their parents [11]. Additionally, where there is a lack of inadequate pension and health insurance for the elders, especially in rural areas and less developed regions (such as western China), more elderly parents have to depend on financial supports from their adult children [32]. However, financial transfers from adult children is not just about the money aspect, it is part of the overall connection between generations. It is important that elderly parents embedded in inter-generational relationship actually enjoy them [27] as this is part of the Chinese traditional cultural of filial piety to take good care of old parents. Higher financial transfers from adult children may reflect a greater personal filial bonds and this may explain the lower risk of depressive symptoms [36]. Emotional support has a large influence on elders’ mental health [37] which may agree with our observation that a higher amount of financial transfers from adult children relates to to fewer depressive symptoms among the elders. Therefore, financial transfers and emotional support to old parents should be encouraged to prevent detoriation of the mental health of older partnes, especially in case of non-co-resident children.

For more robust results, we controlled for demographic, socioeconomic, heath status and community-level variables of the mid-aged and elderly parents; rural-urban areas and seven geographic regions of China were all involved in the analysis. Consistent with the literatures [20, 23, 38], aging (aged 62–69 compare with aged 45–56 years old), being females, having poorer health status are risk factors of depressive symptoms; while living with a child or spouse, having better socioeconomic status and living in well developed areas are protective factors. Intergenerational relationships are maintained by reciprocity [22], and thus taking care of grandchildren might be a kind of life burden for old parents which is a risk factor of having depressive symptoms. Besides, among the aged 45–56, 56–62, 62–69 years old groups, the percent of taking care of grandchildren were 44.75, 54.91, 47.68 and 21.4%. We thus suppose the aged 69 years old and over group compare with the groups with younger age might have less life burden such as taking care of grandchildren. As a result, the parents aged 69 years old and over may experience fewer depressive symptoms.

Several limitations need to be considered in the interpretion of our results. First, since this study was based on cross-sectional data, the causality between inter-generational financial transfers and depressive symptoms could not be determined. Second, in CHARLS CESD-10 questions and answers were based on self-report andthus reported bias may exist in this study. Third, because of too few financial transfers from parents to children, it’s impossible to adjust for these in this study. To explore this problem, we adjusted for taking care of grandchildren, which is a main kind of transfer giving by parents in China, to limit this potential bias.

## Conclusions

Our study revealed that higher amount of financial support from non-co-resident children was positively related to fewer depressive symptoms among the mid-aged and elderly parents in China. Potential policy interventions may need to be explored to promote these and to fill the gaps when financial transfer from adult children decrease.

## Additional file

Additional file 1: CESD-10 questions and answers (DOCX 15 kb)

## Abbreviations

ADL: Aactivities of daily living.
CESD-10: 10-item Center for Epidemiologic Studies–Depression Scale
CHARLS: China Health and Retirement Longitudinal Study
