# Supplementary material for: Financial transfers from adult children and depressive symptoms among mid-aged and elderly residents in China - evidence from the China health and retirement longitudinal study
Source: BMC Public Health. 2018 Jul 16;18:882. doi: 10.1186/s12889-018-5794-x (PMC6048803; doi:10.1186/s12889-018-5794-x)
Supplement: Supplementary file 1 — CESD-10 questions and answers (DOCX 15 kb) [file 12889_2018_5794_MOESM1_ESM.docx]

| **Additional file 1 ：** CESD-10 questions and answers |
| --- |
| The 10 items below refer to how you have felt and behaved during the last week. Choose the appropriate response. |
| Questions |
| 1. I was bothered by things that don’t usually bother me. |
| 1. I had trouble keeping my mind on what I was doing. |
| 1. I felt depressed. |
| 1. I felt everything I did was an effort. |
| 1. I felt hopeful about the future. （positive one） |
| 1. I felt fearful. |
| 1. My sleep was restless. |
| 1. I was happy. （positive one） |
| 1. I felt lonely. |
| 1. I could not get “going”. |
| Answers |
| 1. Rarely or none of the time (< 1 day) 2. Some or a little of the time (1-2 days) 3. Occasionally or a moderate amount of the time (3-4 days) 4. Most or all of the time (5-7 days) |
